# Performance of Juglone as a Natural Extract for Inhibiting SRB-Induced Corrosion of Q235 Steel in Seawater

**DOI:** 10.3390/microorganisms14050966

**Published:** 2026-04-24

**Authors:** Ke Wang, Jie Zhang, Hui Zhang, Xinru Ge, Mathivanan Krishnamurthy, Ruiyong Zhang, Xinyi Zeng, Zhenhua Yu

**Affiliations:** 1 State Key Laboratory of Advanced Marine Materials, Key Laboratory of Marine Environmental Corrosion and Bio-Fouling, Institute of Oceanology, Chinese Academy of Sciences, Qingdao 266071, China; wangke232@mails.ucas.ac.cn (K.W.); zhangjie@qdio.ac.cn (J.Z.); zhanghui951227@163.com (H.Z.);; 2 Qingdao Municipal Center for Disease Control and Prevention, Qingdao 266000, China; 3 Qingdao Institute of Preventive Medicine, Qingdao 266000, China; 4 University of Chinese Academy of Sciences, 19 (Jia) Yuquan Road, Beijing 100049, China; 5 Department of Biomaterials, Saveetha Dental College and Hospitals, Saveetha Institute of Medical and Technical Sciences, Saveetha University, Chennai 600077, India

**Keywords:** sulfate-reducing bacteria (SRB), juglone, microbiologically influenced corrosion (MIC), marine corrosion, green inhibitor

## Abstract

Juglone (5-hydroxy-1,4-naphthalenedione), a natural compound derived from walnut husks, was investigated as a sustainable dual-function antibacterial agent and corrosion inhibitor for Q235 steel in sulfate-reducing bacteria (SRB)-containing seawater. Juglone was synthesized via an improved one-pot method, and its performance was evaluated through antibacterial assays, weight loss measurements, surface characterization (SEM, XPS, XRD), and electrochemical techniques (EIS, PDP). Juglone exhibited potent antibacterial activity against *Desulfovibrio* sp., with a minimum inhibitory concentration (MIC) of 40 mg/L. At 20 mg/L (0.5 MIC) and 40 mg/L (1 MIC), it effectively suppressed bacterial growth and metabolism, mitigating corrosion. At 80 mg/L (2 MIC), a dual-action mechanism was observed: strong antibacterial effect combined with chemical reaction with H_2_S, a corrosive SRB metabolite, forming a protective thiol-containing film on the steel surface. This reduced the corrosion current density from 3.16 × 10^−5^ A/cm^2^ to 7.94 × 10^−7^ A/cm^2^, achieving an inhibition efficiency of 97.5%. Juglone represents a promising green alternative to conventional toxic antibacterial agents, aligning with circular economy principles.

## 1. Introduction

Steel and its alloys serve as fundamental materials for marine engineering structures, including offshore platforms, pipelines, and port facilities. However, these structures are continuously exposed to a harsh corrosive marine environment characterized by high salinity, intense ultraviolet radiation, and complex microbial ecosystems, making them highly susceptible to corrosion damage [1,2]. The economic losses, safety incidents, and maintenance challenges associated with marine corrosion are substantial, necessitating continuous innovation in protective strategies [3]. Among various degradation mechanisms, microbiologically influenced corrosion (MIC) represents a major form of deterioration, where microorganisms directly or indirectly participate in electrochemical corrosion processes, often leading to premature failure of marine infrastructure [4,5].

Microorganisms, as the most abundant decomposers in marine ecosystems, readily colonize material surfaces and form biofilms. These biofilms create unique microenvironments at the metal–solution interface, where parameters such as pH, dissolved oxygen concentration, and chemical gradients differ significantly from those in the surrounding bulk seawater [6,7]. Such localized conditions can dramatically accelerate the corrosion of metallic substrates, shortening service life and posing serious safety risks. Among the diverse microbial communities, sulfate-reducing bacteria (SRB) are recognized as the primary culprits in anaerobic MIC, accounting for over 50% of documented MIC cases [8,9]. The mechanisms by which SRB accelerate steel corrosion are well established: (1) direct electron transfer (DET), whereby bacteria extract electrons directly from metallic iron to support their metabolic respiration, leading to anodic dissolution (Fe → Fe^2+^ + 2e^−^), a process commonly referred to as electrical MIC (E-MIC) [10,11]; and (2) metabolite-induced corrosion, where corrosive metabolic byproducts, particularly hydrogen sulfide (H_2_S), react aggressively with the steel surface, forming iron sulfide (FeS) layers that can further catalyze localized corrosion [12,13].

To mitigate SRB-induced corrosion, strategies typically focus on interfering with these pathways, primarily by inhibiting bacterial metabolic activity and growth (e.g., through bacterial killing or preventing attachment) or by neutralizing corrosive metabolites. The application of biocides represents a common and effective approach capable of addressing both aspects. However, traditional biocides, such as tributyltin (TBT) and tetrakis(hydroxymethyl)phosphonium sulfate (THPS), while effective, suffer from significant drawbacks, including high environmental toxicity, bioaccumulation, high dosage requirements, and elevated costs [14,15]. These limitations have generated an urgent need for the development of novel, efficient, and environmentally friendly “green” biocides and corrosion inhibitors.

In recent years, natural products derived from biomass have emerged as ideal sources for green corrosion inhibitors due to their advantages of biodegradability, low toxicity, low cost, and widespread availability [16,17]. Numerous plant-derived extracts have been explored as green corrosion inhibitors for steel in marine environments, including Andrographis paniculata essential oil, which demonstrated excellent inhibition performance in artificial seawater [18]. Additionally, synthetic inhibitors designed following green chemistry principles have shown promise. For instance, Haque et al. (2023) developed water-soluble sodium L-2-(1-imidazolyl) alkanoic acids from natural amino acid derivatives, which exhibited effective corrosion inhibition for mild steel in artificial seawater via protective film formation [19]. Among these, walnut *(Juglans regia* L.) is a major economic crop that generates substantial quantities of husk waste annually, which is often discarded as solid waste and poses certain environmental challenges. Notably, walnut husks are rich in bioactive components, including phenolic compounds, fatty acids, and aromatic compounds [20]. One of the most prominent active constituents is juglone (5-hydroxy-1,4-naphthoquinone), whose insecticidal, anticancer, and anti-inflammatory properties have been extensively studied [21,22]. Juglone was first isolated from walnut husks in the 1850s, and its unique chemical structure, which possesses both oxidative and reductive properties, has attracted considerable research interest. Of particular relevance, juglone is known to react with sulfhydryl groups; for instance, its reaction with hydrogen sulfide has been documented for decades [23]. Despite its well-recognized antimicrobial activity and chemical reactivity that make it highly promising for MIC prevention, the application of juglone as a dual-function biocide and corrosion inhibitor remains rarely reported.

Beyond its technical merits, the utilization of juglone carries significant socioeconomic and environmental importance. Socially, valorizing walnut husk waste into a high-value chemical like juglone promotes a circular economy, addressing agricultural waste management issues while providing sustainable solutions for industrial challenges such as corrosion [24,25]. This aligns with global sustainability initiatives, including the United Nations Sustainable Development Goals, by fostering responsible consumption and production patterns [26]. Economically, with over 3.5 million tons of walnuts produced annually worldwide, the associated husk waste represents a vast and underutilized resource [27]. Transforming this waste stream into juglone offers a cost-effective alternative to expensive synthetic biocides, potentially reducing maintenance costs for critical marine infrastructure while creating new revenue streams for agricultural communities in major walnut-producing regions such as China and the United States [28,29]. The market for green corrosion inhibitors is expanding rapidly, driven by stringent environmental regulations, with some estimates projecting the market for green alternatives to exceed $2 billion by 2028 [30]. This creates a substantial market opportunity for juglone-based products, particularly in the offshore oil and gas and marine transport sectors [31].

In view of this, the present study aims to explore the potential of juglone as a green biocide for inhibiting SRB-induced corrosion of Q235 steel in marine environments. High-purity juglone was synthesized using an improved one-pot method, and its antibacterial activity against a marine SRB strain (*Desulfovibrio* sp.) was systematically evaluated. The corrosion inhibition performance on Q235 steel was comprehensively assessed through weight loss measurements, surface analysis techniques (SEM, CLSM, XPS, XRD), and electrochemical methods (OCP, EIS, PDP). The results demonstrate that juglone not only effectively kills SRB but also mitigates corrosion through a dual-action mechanism involving antibacterial activity and in situ formation of a protective film via reaction with H_2_S. This work provides a promising and sustainable alternative to conventional toxic biocides for controlling MIC in marine applications.

## 2. Experimental

### 2.1. Materials and Bacterial Cultivation

Q235 carbon steel was used as the substrate material for all experiments. The chemical composition (wt.%) of the steel was as follows: C 0.19, Mn 0.55, Si 0.28, P 0.037, S 0.027, and Fe as the balance. For weight loss measurements, steel coupons with dimensions of 5.0 cm × 3.0 cm × 0.2 cm were employed. For electrochemical tests, steel coupons were embedded in epoxy resin, leaving an exposed working area of 1.0 cm × 1.0 cm. Prior to each experiment, the steel surfaces were sequentially ground with silicon carbide (SiC) abrasive papers from 400 to 2000 grit, ultrasonically cleaned in anhydrous ethanol for 10 min, dried under a stream of high-purity nitrogen, and finally sterilized under ultraviolet (UV) light for 30 min.

The sulfate-reducing bacterium (SRB) strain, identified as *Desulfovibrio* sp., was isolated from sediment collected in the South China Sea. The strain was cultivated in modified Postgate’s C (PGC) medium under anaerobic conditions at 30 °C for 3–5 days. The pH of the medium was adjusted to 7.2 ± 0.2 using 0.1 mol/L NaOH solution. Prior to inoculation, the medium was autoclaved at 121 °C for 20 min. After cooling, 0.1% (*w*/*v*) L-cysteine was added as an oxygen scavenger to maintain anaerobic conditions. All chemicals used in this study were of analytical grade.

### 2.2. Synthesis of Juglone

**Scheme 1:**
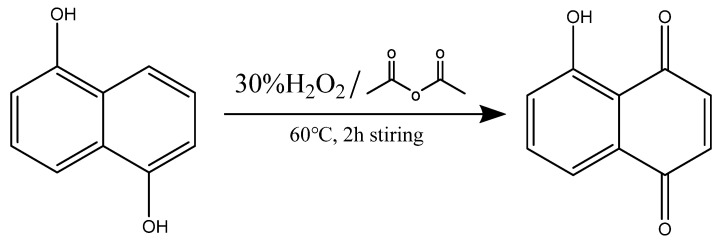
Synthesis route of Juglone.

Juglone (5-hydroxy-1,4-naphthoquinone) was synthesized using a modified one-pot method based on previously reported procedures (Scheme 1) [32]. Briefly, 10 mL of 30% hydrogen peroxide (89 mmol) was cooled in an ice–water bath. Acetic anhydride (5 mL, 46.7 mmol) was added dropwise with continuous stirring, maintaining the reaction temperature below 30 °C. The mixture was stirred for 4 h. Subsequently, 1,5-dihydroxynaphthalene (1 g, 6.25 mmol) dissolved in 20 mL of a dichloromethane:methanol (1:1, *v*/*v*) mixture was added dropwise, keeping the temperature below 60 °C. The reaction was stirred for an additional 2 h. After completion of the reaction, as confirmed by thin-layer chromatography (TLC), the mixture was poured into 60 mL of ice water. The resulting precipitate was collected by vacuum filtration. The crude product was purified by column chromatography on silica gel using a hexane:ethyl acetate (6:1, *v*/*v*) mixture as the eluent. The purified product was dried under an infrared lamp, yielding 960 mg of an orange solid (yield: 83.9%). The structure of the synthesized juglone was confirmed by Fourier-transform infrared (FTIR) spectroscopy.

### 2.3. Antibacterial Activity Assays

The antibacterial activity of juglone against *Desulfovibrio* sp. was evaluated by monitoring bacterial growth and metabolic activity. Juglone was first dissolved in sterile PGC medium containing 0.05% (*v*/*v*) dimethyl sulfoxide (DMSO) as a co-solvent to prepare stock solutions. The stock solutions were then diluted to obtain final working concentrations of 5, 10, 20, 30, 40, 50, 60, 80, 100, and 120 mg/L. An activated SRB culture was diluted to an initial concentration of approximately 5 × 10^6^ colony-forming units per milliliter (CFU/mL) and inoculated into the media containing different juglone concentrations. A culture without juglone served as the biotic control, and sterile medium served as the abiotic control. After 7 days of anaerobic incubation at 30 °C, bacterial growth was estimated by measuring the optical density at 600 nm (OD_600_) using a UV-Vis spectrophotometer. The effect of juglone on SRB metabolism was assessed by measuring the concentration of sulfide, a key metabolic byproduct, using the methylene blue spectrophotometric method. Briefly, sulfide concentration was determined by mixing the culture supernatant with a mixed diamine reagent and measuring the absorbance at 665 nm, according to the standard procedure described by Cline [33]. All experiments were performed in triplicate.

It should be noted that OD_600_ measurements reflect total biomass (including both viable and non-viable cells) and may be influenced by FeS precipitates formed during SRB metabolism [34]. In this study, OD_600_ was used as a semi-quantitative indicator of bacterial growth inhibition, complemented by visual inspection of culture turbidity/FeS precipitation and direct SEM observation of cell morphology, to provide a comprehensive assessment of juglone’s antibacterial effect.

### 2.4. Minimum Inhibitory Concentration (Mic)

The minimum inhibitory concentration (MIC) of juglone against *Desulfovibrio* sp. was defined as the lowest concentration that resulted in over 90% inhibition of bacterial growth compared to the juglone-free control. The MIC was determined using the micro-broth dilution method in 96-well plates [14]. The same concentration gradient of juglone (5 to 120 mg/L) as described in Section 2.3 was applied. After 7 days of anaerobic incubation at 30 °C, the wells were visually inspected for turbidity and the formation of black FeS precipitates. The OD_600_ of each well was also measured to quantitatively determine the inhibition of bacterial growth. All measurements were performed in triplicate.

The MIC value was corroborated by visual inspection of culture turbidity and the absence of black FeS precipitates, both indicative of suppressed SRB growth and metabolic activity.

### 2.5. Weight Loss Experiment

Pre-weighed Q235 steel coupons were immersed in sterile PGC medium inoculated with SRB, containing juglone at concentrations of 0 (control), 0.5 MIC (20 mg/L), 1 MIC (40 mg/L), and 2 MIC (80 mg/L). After 15 days of immersion at 30 °C, the coupons were retrieved. The surface biofilm and corrosion products were removed according to the standard procedure ASTM G1-03 [35], using a cleaning solution composed of 500 mL hydrochloric acid (HCl), 50 g stannous chloride (SnCl_2_), and 20 g antimony trioxide (Sb_2_O_3_), diluted to a final volume of 1000 mL with deionized water. After cleaning, the coupons were rinsed with deionized water and anhydrous ethanol, dried under a stream of nitrogen, and reweighed. The corrosion rate (v_corr_) was calculated using the following equation:

$$v_{corr}=\;\frac{8.76\times 10^{4}\times \Delta m}{\rho At}$$

where v_corr_ is the corrosion rate (mm/a), Δm is the mass loss (g), ρ is the density of the steel (7.85 g/cm^3^), A is the exposed surface area (cm^2^), and t is the immersion time (h). All weight loss experiments were performed in triplicate.

### 2.6. Surface Characterization

After 14 days of immersion, steel coupons were retrieved for surface analysis. For scanning electron microscopy (SEM), coupons were fixed in 2.5% (*v*/*v*) glutaraldehyde solution for 2 h at 4 °C, followed by sequential dehydration in ethanol solutions of increasing concentrations (25%, 50%, 70%, 80%, 90%, and 100% *v*/*v*) for 10 min each. After drying and gold sputter coating, the surface morphology of the biofilm and corrosion products was observed using a field emission scanning electron microscope (FE-SEM, FEI Quanta 250, FEI Company, Brno, Czech Republic) operated at an accelerating voltage of 10 kV.

The topography and depth of corrosion pits on cleaned coupons were measured using a three-dimensional confocal laser scanning microscope (CLSM, Lext OLS5000, Evident Corporation, Tokyo, Japan). The crystalline phases of the corrosion products were analyzed by X-ray diffraction (XRD, D/max-Ultima IV, Rigaku Corporation, Tokyo, Japan) with Cu Kα radiation (λ = 0.15418 nm) at a scanning rate of 5°/min in the 2θ range of 10° to 80°. The elemental composition and chemical states of the corrosion products were determined by X-ray photoelectron spectroscopy (XPS, K-Alpha, Thermo Fisher Scientific, Waltham, MA, USA) using monochromatic Al Kα radiation (hν = 1486.6 eV). The binding energy scale was calibrated using the C 1s peak at 284.8 eV.

### 2.7. Electrochemical Measurements

Electrochemical tests were conducted using a P4000A electrochemical workstation (Gamry Instruments, Warminster, PA, USA) in a standard three-electrode cell configuration. The Q235 steel coupon with an exposed area of 1.0 cm^2^ served as the working electrode, a saturated calomel electrode (SCE) as the reference electrode, and a platinum sheet (1.0 cm × 1.0 cm) as the counter electrode. The electrolyte was SRB-inoculated PGC medium containing juglone at concentrations of 0, 0.5 MIC, 1 MIC, and 2 MIC. All electrochemical tests were performed at 30 °C, and each experiment was repeated three times to ensure reproducibility.

Open circuit potential (OCP) was monitored continuously for 14 days to evaluate the evolution of the corrosion potential over time. Electrochemical impedance spectroscopy (EIS) was performed at the OCP using a sinusoidal perturbation of 10 mV (peak-to-peak) over a frequency range from 10^5^ Hz to 10^−2^ Hz, with 10 points per decade. The EIS data were fitted to equivalent electrical circuits (EECs) using ZSimpWin software (Version 3.60). Potentiodynamic polarization (PDP) curves were measured after 14 days of immersion by scanning the potential from −250 mV to +250 mV versus OCP at a scan rate of 0.167 mV/s. The corrosion current density (i_corr_) was determined by Tafel extrapolation of the cathodic and anodic branches. The corrosion inhibition efficiency (η) was calculated from the PDP results using the following equation:

$$\eta =\;\frac{i_{corr,0}-i_{corr}}{i_{corr,0}}\times 100\%$$

where i_corr,0_ and i_corr_ are the corrosion current densities for the unprotected and protected working electrodes, respectively.

### 2.8. Data Availability and Statistical Analysis

All experiments were performed in triplicate, and the results are presented as mean ± standard deviation (SD). Statistical analysis was performed using one-way analysis of variance (ANOVA), and a *p*-value of less than 0.05 was considered statistically significant. The datasets generated and analyzed during the current study are available from the corresponding author upon reasonable request.

## 3. Results and Discussion

### 3.1. Antibacterial Activity of Juglone

**Figure 1:**
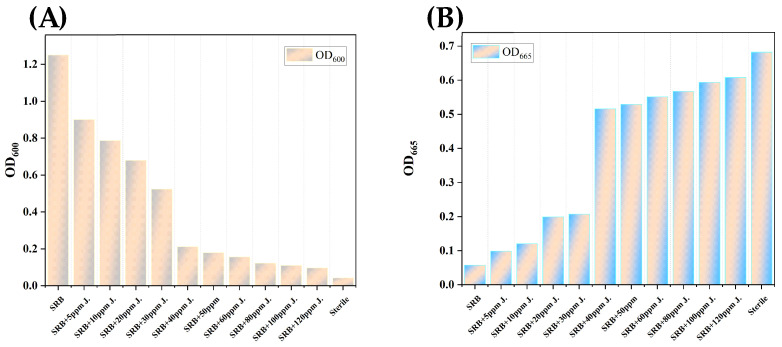
(**A**) OD600 values of SRB cultures after 7 days of incubation at different juglone (J.) concentrations. (**B**) UV-Vis absorbance spectra at 665 nm (methylene blue assay) indicating relative sulfide concentrations in SRB cultures after 7 days of incubation at different juglone (J.) concentrations.

The antibacterial activity of juglone against *Desulfovibrio* sp. was evaluated by monitoring bacterial growth (OD_600_) and sulfide production, a key metabolic byproduct of SRB, after 7 days of anaerobic incubation. As shown in Figure 1A, the biotic control group (0 mg/L juglone) exhibited an OD_600_ value exceeding 1.0, indicating robust bacterial proliferation. The addition of 5 mg/L juglone resulted in a slight reduction in OD_600_ to 0.894, suggesting a marginal inhibitory effect. With increasing juglone concentration, a clear dose-dependent antibacterial response was observed. At 30 mg/L, the OD_600_ decreased to 0.503, reflecting a substantial reduction in viable bacterial populations. A sharp decline to 0.218 was recorded at 40 mg/L, indicating a pronounced antibacterial effect.

Consistent with the growth inhibition trends, sulfide production was also significantly suppressed by juglone, as determined by the methylene blue spectrophotometric method (Figure 1B). In the biotic control, the absorbance at 665 nm was 0.057, corresponding to high sulfide production. The addition of juglone at concentrations ranging from 5 to 30 mg/L increased the absorbance to values below 0.2, indicating partial but incomplete inhibition of SRB metabolic activity. Notably, at a juglone concentration of 40 mg/L, the absorbance surged to 0.55, representing an approximately threefold increase compared to the biotic control. This sharp increase confirms that juglone effectively suppressed SRB metabolism, leading to the accumulation of residual sulfide in the culture medium. These results collectively demonstrate that juglone exhibits potent and dose-dependent antimicrobial activity against *Desulfovibrio* sp., effectively inhibiting both bacterial growth and metabolic activity.

### 3.2. Minimum Inhibitory Concentration (Mic)

**Figure 2:**
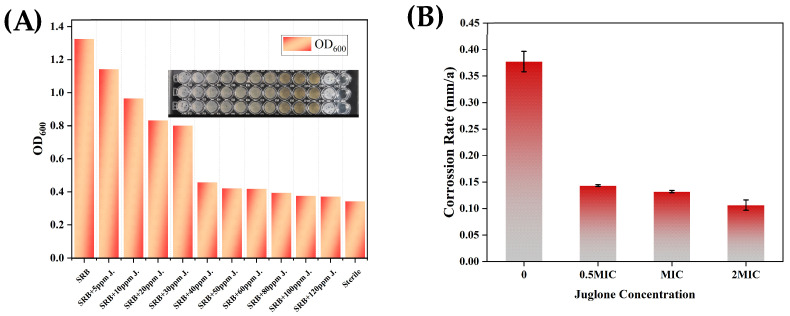
(**A**) OD600 values from the 96-well MIC assay after 7 days of incubation. (**B**) Corrosion rates of Q235 steel coupons after 15 days of immersion in SRB-inoculated medium with different concentrations of juglone.

The minimum inhibitory concentration (MIC) of juglone against *Desulfovibrio* sp. was determined using the micro-broth dilution method. After 7 days of anaerobic incubation, visual inspection of the 96-well plate revealed that wells containing juglone concentrations below 40 mg/L exhibited turbidity and the formation of black FeS precipitates, indicative of active SRB growth and metabolism. In contrast, wells containing juglone at concentrations of 40 mg/L and above remained clear, with no visible FeS precipitates. Quantitative analysis of OD_600_ values (Figure 2A) confirmed that the growth inhibition rate exceeded 90% at a juglone concentration of 40 mg/L compared to the biotic control. Accordingly, the MIC of juglone against the marine *Desulfovibrio* sp. strain was determined to be 40 mg/L.

### 3.3. Weight Loss and Corrosion Rate

The corrosion inhibition performance of juglone was further evaluated through weight loss measurements after 15 days of immersion in SRB-inoculated medium. As shown in Figure 2B, the Q235 steel coupon immersed in the biotic control medium (0 mg/L juglone) exhibited a severe corrosion rate of 0.377 ± 0.02 mm/a. In contrast, the addition of juglone resulted in a significant reduction in corrosion rate in a dose-dependent manner. At a concentration of 20 mg/L (0.5 MIC), the corrosion rate decreased to 0.145 ± 0.01 mm/a. Further increases in juglone concentration to 40 mg/L (1 MIC) and 80 mg/L (2 MIC) led to corrosion rates of 0.089 ± 0.01 mm/a and 0.041 ± 0.005 mm/a, respectively. These results clearly demonstrate that juglone provides excellent protection against SRB-induced corrosion, with the protective effect increasing proportionally with inhibitor concentration.

### 3.4. Surface Analysis

**Figure 3:**
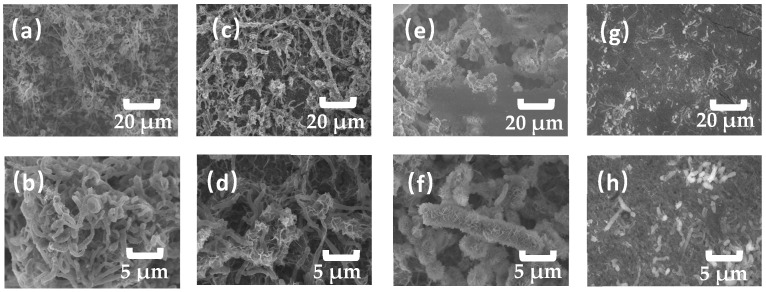
SEM micrographs of Q235 steel surfaces after 14 days of immersion in SRB-inoculated medium with: (**a**,**b**) 0 mg/L, (**c**,**d**) 0.5 MIC, (**e**,**f**) 1 MIC, and (**g**,**h**) 2 MIC of juglone.

Scanning electron microscopy (SEM) was employed to examine the surface morphology of Q235 steel coupons after 14 days of immersion under different conditions (Figure 3). In the absence of juglone (Figure 3a,b), the steel surface was completely covered by a dense and mature biofilm composed of SRB cells embedded in a matrix of extracellular polymeric substances (EPS) [36,37]. This biofilm structure is known to create aggressive localized microenvironments that accelerate corrosion. Upon the addition of juglone at a sub-inhibitory concentration of 20 mg/L (0.5 MIC, Figure 3c,d), a noticeable reduction in sessile bacterial density was observed. Flocculent precipitates were visible on bacterial cell surfaces, suggesting compromised membrane integrity and leakage of cytoplasmic contents, consistent with previous reports on the antimicrobial mechanisms of juglone [38,39].

At a concentration of 40 mg/L (1 MIC, Figure 3e,f), the antibacterial effect was markedly amplified. Bacterial cells lost their characteristic rod-like morphology and appeared as ruptured and disintegrated cellular debris. Notably, residual cellular matter appeared to be encapsulated within a thin, poorly conductive film, which may be attributed to the interaction of juglone with bacterial metabolites or EPS, potentially forming a passivating layer on the steel surface [40]. At the highest tested concentration of 80 mg/L (2 MIC, Figure 3g,h), the steel surface exhibited a nearly pristine morphology, with only sparse and ill-defined bacterial remnants. This observation indicates that at higher concentrations, juglone exerts a rapid and potent antibacterial action that effectively prevents biofilm formation and subsequent MIC.

### 3.5. Composition of Corrosion Products

**Figure 4:**
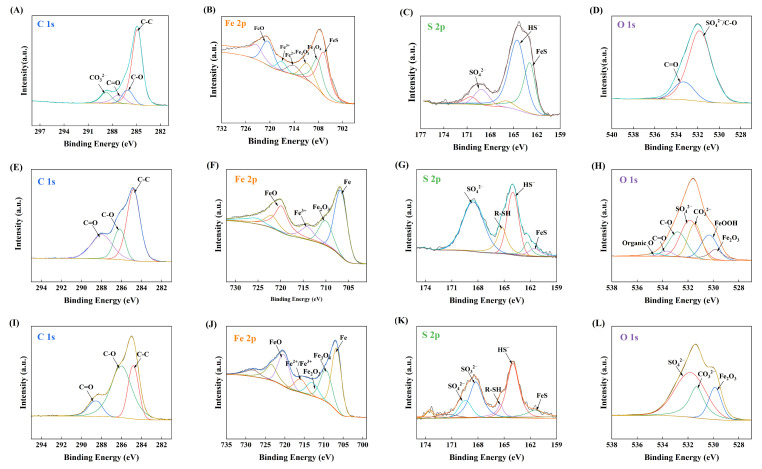
High-resolution XPS spectra of C 1s, O 1s, Fe 2p, and S 2p for corrosion products after 14 days of immersion with: (**A**–**D**) 0 mg/L, (**E**–**H**) 0.5 MIC, and (**I**–**L**) 1 MIC of Juglone.

The chemical composition of corrosion products formed on Q235 steel after 14 days of immersion was analyzed using X-ray photoelectron spectroscopy (XPS) and X-ray diffraction (XRD). The relative atomic percentages of elements on the surface, derived from XPS analysis, are summarized in Table S1 (Supplementary Materials). In the SRB control group, the surface exhibited a high carbon content (55.17%), attributed to the thick biofilm and EPS [7]. The high-resolution S 2p spectrum (Figure 4) revealed a distinct peak at 162.6 eV, characteristic of iron sulfide (FeS), a typical corrosion product associated with SRB metabolism [41]. The Fe 2p spectrum further confirmed the presence of both iron oxides and FeS.

Upon the addition of juglone, distinct changes in the composition of the corrosion products were observed. At 0.5 MIC (20 mg/L) and 1 MIC (40 mg/L), the carbon and sulfur contents decreased, while the oxygen and iron contents increased. Notably, in the 1 MIC sample, the FeS peak was absent in the Fe 2p spectrum, and the S 2p spectrum revealed new peaks not present in the control. Besides a weak sulfide peak, prominent peaks appeared at 168.8–169.2 eV (SO_4_^2−^), 165.3 eV (R–SH), and 164.1 eV (HS^−^) [42]. The appearance of the R–SH (thiol) peak is particularly significant, as it suggests a chemical reaction between juglone and H_2_S, a primary SRB metabolic product, resulting in the formation of a thiol-containing derivative. This finding supports the hypothesis that juglone can scavenge H_2_S and generate a protective film on the steel surface.

**Figure 5:**
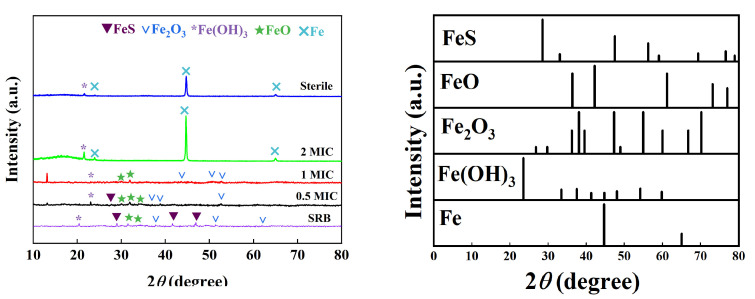
XRD patterns of corrosion products on Q235 steel after 14 days of immersion under different conditions.

XRD analysis (Figure 5) corroborated the XPS findings. The abiotic control and the 2 MIC (80 mg/L) sample exhibited only peaks corresponding to the iron substrate, indicating minimal corrosion. In contrast, the SRB control sample displayed strong characteristic peaks at 29.1° and 47.9°, confirming the formation of FeS. For the 0.5 MIC sample, a weak FeS peak was detected at 28.6°, suggesting partial inhibition of FeS formation. In the 1 MIC sample, FeS peaks were undetectable, and peaks corresponding to iron oxides (FeO and Fe_2_O_3_) were observed at 32.1° and 53.2°. These results collectively indicate that juglone effectively inhibits the formation of corrosive FeS, likely through both its antibacterial activity and its ability to chemically interact with H_2_S, thereby promoting the formation of a more protective iron oxide-rich surface film.

### 3.6. Electrochemical Measurements

#### 3.6.1. Open Circuit Potential (OCP)

**Figure 6:**
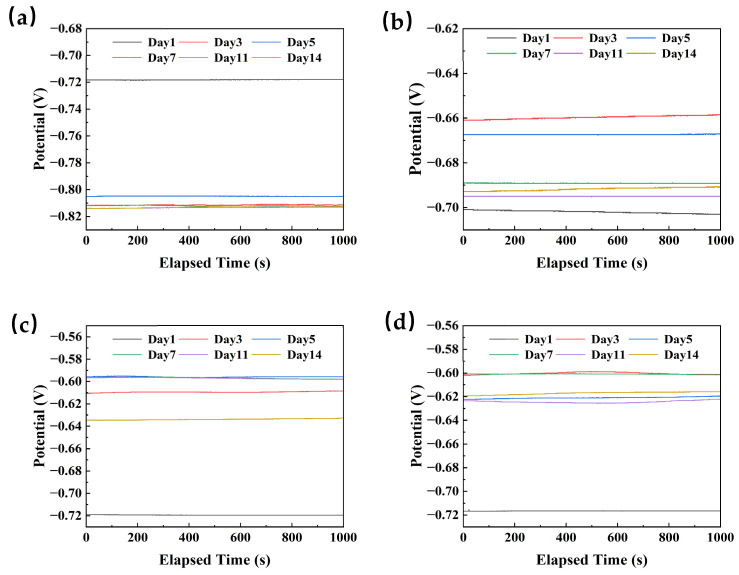
OCP evolution of Q235 steel over 14 days in SRB-inoculated medium with: (**a**) 0 mg/L, (**b**) 0.5 MIC, (**c**) 1 MIC, and (**d**) 2 MIC of juglone.

The evolution of open circuit potential (OCP) over 14 days of immersion is presented in Figure 6. In the SRB control system (Figure 6a), the OCP rapidly shifted to more negative values, stabilizing around −0.81 V after 7 days. This negative shift is characteristic of active SRB-induced corrosion, driven by the metabolic activity of the biofilm that accelerates anodic iron dissolution [43]. In contrast, the addition of juglone resulted in a positive (noble) shift in OCP. At concentrations of 20 mg/L (0.5 MIC), 40 mg/L (1 MIC), and 80 mg/L (2 MIC), the OCP reached maximum values of −0.661 V, −0.596 V, and −0.601 V, respectively, within the first 5 days. The OCP in the 0.5 MIC system began to decrease after day 7, likely due to the gradual consumption and depletion of juglone, allowing corrosion to resume. Notably, the 2 MIC system maintained a relatively stable and noble OCP around −0.62 V throughout the 14-day immersion period, suggesting the formation of a stable and effective protective layer on the steel surface. This observation is consistent with the surface analysis results, which indicated a nearly pristine surface at this concentration.

#### 3.6.2. Electrochemical Impedance Spectroscopy (EIS)

**Figure 7:**
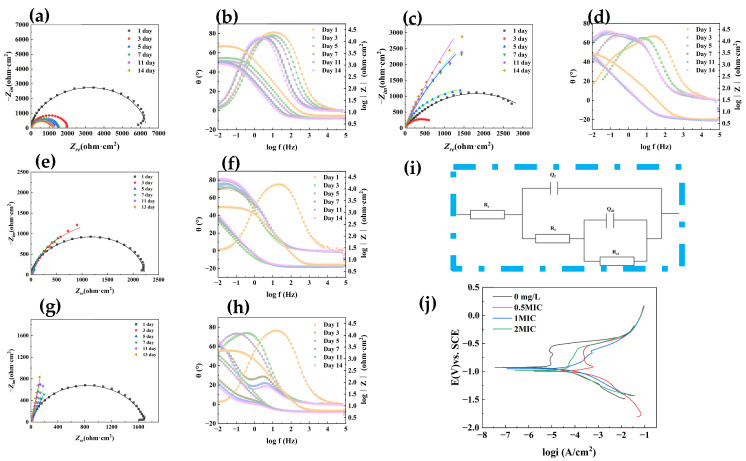
EEC (**i**), PDP (**j**), Nyquist and Bode plots for Q235 steel after different immersion times in SRB-inoculated medium with: (**a**,**b**) 0 mg/L, (**c**,**d**) 0.5 MIC, (**e**,**f**) 1 MIC, and (**g**,**h**) 2 MIC of juglone.

Electrochemical impedance spectroscopy (EIS) was employed to investigate the evolution of the steel/electrolyte interface over time. The Nyquist and Bode plots, along with the equivalent electrical circuit (EEC) used for fitting, are shown in Figure 7a–h and the equivalent electrical circuit (EEC) used for fitting the EIS data is shown in Figure 7i. The EEC model (Figure 7i) consisted of solution resistance (R_s_), film resistance (R_f_) with its associated constant phase element (Q_f_), charge transfer resistance (R_ct_), and double-layer constant phase element (Q_dl_). R_ct_ is inversely proportional to the corrosion rate and serves as a key indicator of inhibition performance.

In the SRB control system (Figure 7a,b), the diameter of Nyquist plots decreased progressively over time, and the R_ct_ values dropped significantly, reflecting an acceleration of corrosion due to SRB activity. A slight increase in R_ct_ observed after day 7 may be attributed to the thickening of the biofilm and corrosion product layer, which partially hindered mass transport [44].

Upon the addition of juglone, the corrosion process was significantly impeded. In the 0.5 MIC system (Figure 7c,d), R_ct_ initially decreased but then rose sharply to 3581 Ω·cm^2^ on day 5, indicating that juglone began to inhibit corrosion after an initial adaptation period. At 1 MIC (Figure 7e,f), R_ct_ increased steadily from day 1, demonstrating immediate and sustained inhibition, although a slight decrease was observed after day 11, likely due to inhibitor depletion. Most notably, in the 2 MIC system (Figure 7g,h), the impedance modulus and R_ct_ values increased continuously and dramatically throughout the 14-day immersion period, reaching exceptionally high values (R_ct_ > 10^4^ Ω·cm^2^). The Bode plots for the 2 MIC system revealed the emergence of a second time constant at higher frequencies, and the EEC fitting required the inclusion of a film resistance (R_f_). These observations strongly support the formation of a dense, stable, and highly protective film on the steel surface, which acts as an effective barrier against corrosive species.

#### 3.6.3. Potentiodynamic Polarization (PDP)

Potentiodynamic polarization (PDP) curves measured after 14 days of immersion are presented in Figure 7j, and the corresponding electrochemical parameters are summarized in Table S2 (Supplementary Materials). In the SRB control group, the corrosion current density (i_corr_) was 3.16 × 10^−5^ A/cm^2^. The addition of juglone resulted in a significant reduction in i_corr_ in a dose-dependent manner. At 0.5 MIC, 1 MIC, and 2 MIC, i_corr_ decreased to 6.31 × 10^−6^, 1.58 × 10^−6^, and 7.94 × 10^−7^ A/cm^2^, respectively. The corresponding corrosion inhibition efficiency (η), calculated from i_corr_ values, reached an outstanding 97.5% at a juglone concentration of 80 mg/L (2 MIC).

The corrosion potential (E_corr_) exhibited a slight anodic shift upon the addition of juglone, with all shifts remaining less than 85 mV, indicating that juglone acts as a mixed-type inhibitor [40,45]. Both the anodic and cathodic branches of the polarization curves were suppressed, suggesting that juglone simultaneously impedes the anodic dissolution of iron and the cathodic hydrogen evolution reaction. This mixed-type inhibition behavior is consistent with the formation of a protective film on the steel surface, which blocks active sites for both anodic and cathodic reactions.

### 3.7. Mechanistic Discussion: Dual-Action Inhibition by Juglone

Based on the comprehensive experimental results presented above and in conjunction with relevant literature, a dual-action mechanism is proposed for juglone in inhibiting SRB-induced corrosion of Q235 steel. This mechanism encompasses direct antibacterial activity against SRB cells and the chemical interaction with SRB metabolic byproducts to form a protective film on the metal surface.

#### 3.7.1. Antibacterial Mechanism: Membrane Disruption and Oxidative Stress

The SEM micrographs (Figure 3) clearly demonstrate that juglone induces dramatic morphological changes in SRB cells, transforming them from intact rod-shaped structures to collapsed and ruptured debris. This observation indicates that the structural integrity of the cell wall and cell membrane is severely compromised. Juglone possesses an amphipathic molecular structure, characterized by a hydrophobic naphthoquinone ring and hydrophilic hydroxyl and carbonyl moieties. This amphipathic nature facilitates its intercalation into the bacterial cell membrane, disrupting membrane fluidity and integrity, leading to leakage of cytoplasmic contents and cell death. This mode of action is analogous to that of antimicrobial peptides, which disrupt membranes through electrostatic and hydrophobic interactions [38,39].

While quinone compounds are known to induce oxidative stress via reactive oxygen species (ROS) generation under aerobic conditions [46,47], the strictly anaerobic environment employed in this study limits the relevance of ROS-mediated pathways. Therefore, the pronounced membrane damage observed by SEM (Figure 3) is more reasonably attributed to direct membrane disruption rather than oxidative stress. This interpretation is fully consistent with the visual evidence of collapsed and ruptured bacterial cells in the presence of juglone.

#### 3.7.2. Corrosion Inhibition Mechanism: H_2_S Scavenging and Protective Film Formation

A key finding of this study is the exceptional corrosion inhibition performance of juglone at higher concentrations (2 MIC), a mechanism that extends beyond its antibacterial activity. The detection of a characteristic R–SH (thiol) peak at 165.3 eV in the XPS spectrum of the 1 MIC sample provides critical evidence for this mechanism. We propose that juglone chemically reacts with hydrogen sulfide (H_2_S), a highly corrosive metabolite produced by SRB. This reaction generates a thiol-containing derivative, which subsequently adsorbs onto the steel surface to form a protective film.

**Figure 8:**
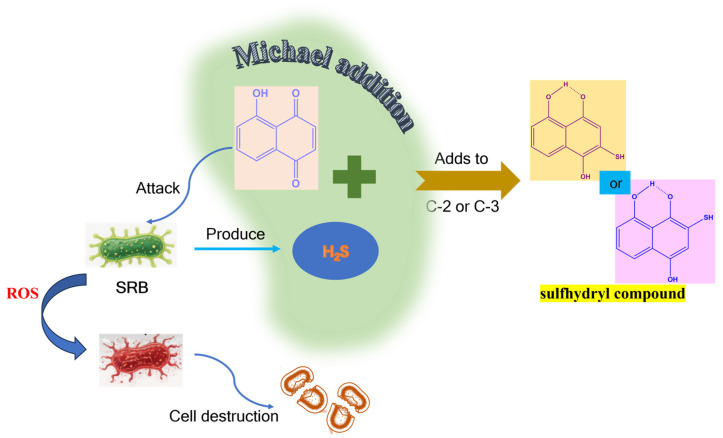
Proposed mechanism for the antibacterial action of juglone and its reaction with H_2_S to form a protective film.

Juglone is classified as an α,β-unsaturated carbonyl compound, in which the carbon–carbon double bond of the quinone ring serves as an electrophilic center susceptible to nucleophilic attack via Michael addition [48]. This reaction is driven by the high electrophilicity of the C2–C3 double bond in the quinone ring, which is activated by the adjacent carbonyl groups. The nucleophilic hydrosulfide ion preferentially adds to the less hindered C3 position, leading to the formation of a thiol-substituted naphthoquinone derivative [49]. The simplest structure consistent with NMR analysis was a sulfur molecule containing sulfur bonded between two juglone molecules at the C-2 or C-3 position, while EPR measurements indicated the presence of a stable semiquinone intermediate with a sulfur substituent [47]. Based on these established findings, we propose that a similar sulfur-containing juglone derivative is generated in our system, which subsequently adsorbs onto the steel surface to form a protective film. Accordingly, we propose that juglone reacts with H_2_S via the pathway illustrated in Figure 8, generating a juglone derivative containing a thiol (–SH) group.

This reaction is of dual significance. First, it directly consumes and scavenges H_2_S from the interfacial microenvironment, eliminating a primary corrosive agent at its source and preventing the formation of iron sulfide (FeS). This is consistent with the disappearance of FeS peaks in the XRD and XPS analyses at higher juglone concentrations. Second, the resulting thiolated derivative is itself a highly effective corrosion inhibitor. Organic molecules containing thiol groups are renowned for their strong adsorption on iron surfaces, as the sulfur atom can form strong coordinate bonds with the vacant d-orbitals of surface iron atoms, establishing a stable chemisorbed film [50,51,52]. Moreover, the derivative retains the original aromatic ring, carbonyl, and hydroxyl groups, which also serve as effective adsorption sites [53]. This multi-functional inhibitor molecule can undergo synergistic, multi-point adsorption onto the steel surface, forming a dense and stable protective barrier.

The electrochemical results provide compelling evidence for the formation of this protective film. The EIS data for the 2 MIC system exhibited a second time constant and required the inclusion of a film resistance (R_f_) in the equivalent circuit model, indicating the development of a distinct surface layer. The continuous increase in Rct and the exceptional inhibition efficiency of 97.5% further corroborate the formation of a highly protective barrier. Therefore, the corrosion inhibition action of juglone can be described as an “intelligent” response: it utilizes a detrimental byproduct of SRB metabolism (H_2_S) as a reactant to generate a more efficacious protective agent in situ. This dual function provides synergistic antibacterial and corrosion inhibition effects.

#### 3.7.3. Green Chemistry Considerations and Comparative Performance

Although the juglone used in this study was synthesized via an organic solvent-based method (Section 2.2) to ensure high purity and yield for fundamental mechanistic investigation, it is important to note that future practical applications can employ greener synthetic approaches. Alternative green synthesis routes for juglone have been reported, including solvent-free polymer-supported synthesis, reactions in ionic liquids, and photooxygenation “on water,” which can achieve comparable yields (up to 81%) while minimizing environmental impact [54]. Furthermore, the dichloromethane and methanol used in the present synthesis can be recovered by standard solvent recycling systems, improving the overall sustainability profile. From a broader perspective, the valorization of walnut husk waste into juglone itself represents a sustainable strategy aligned with circular economy principles, transforming an agricultural byproduct into a high-value corrosion inhibitor.

To contextualize the performance of juglone, it is instructive to compare its inhibition efficiency and effective dosage with those of conventional biocides and other green alternatives. The widely used commercial biocide THPS (tetrakis(hydroxymethyl)phosphonium sulfate) at 100 mg/L achieves approximately 94% reduction in uniform corrosion rate against *Desulfovibrio ferrophilus* on ×80 carbon steel in artificial seawater [55]. Juglone, at a lower concentration of 80 mg/L, delivers a superior inhibition efficiency of 97.5% against *Desulfovibrio* sp. on Q235 steel. Among bio-based inhibitors, a chitosan/lignosulfonate nanocomposite (CS@LS) required 1000 mg/L to achieve 89% inhibition efficiency [56], while a chitosan derivative (CSC-DI) attained 97.9% efficiency at 200 mg/L [57]. The imidazolyl alkanoic acids developed by Haque et al. (2023) [19] demonstrated promising inhibition at moderate concentrations in artificial seawater, and the Andrographis paniculata extract investigated by Kamaruzzaman et al. (2024) [18] exhibited mixed-type inhibition behavior. Collectively, this comparison highlights juglone as a highly potent green inhibitor that achieves state-of-the-art performance at a relatively low dosage, reinforcing its practical potential for marine MIC mitigation.

## 4. Conclusions

In this study, the potential of juglone, a natural compound extracted from walnut husks, as a green dual-function biocide and corrosion inhibitor for mitigating SRB-induced corrosion of Q235 steel in marine environments was systematically investigated. Based on the comprehensive experimental results and mechanistic analysis, the following conclusions can be drawn:

(1) Juglone exhibits potent antibacterial activity against the marine SRB strain *Desulfovibrio* sp., with a minimum inhibitory concentration (MIC) of 40 mg/L. At this concentration, juglone effectively inhibited both bacterial growth and metabolic activity, as evidenced by the significant reduction in OD_600_ values and sulfide production. The antibacterial mechanism involves disruption of cell membrane integrity by the amphipathic juglone molecule.
(2) Juglone provides excellent corrosion protection for Q235 steel in SRB-containing seawater in a dose-dependent manner. Weight loss measurements and electrochemical tests (EIS and PDP) consistently demonstrated that the corrosion inhibition efficiency increased with increasing juglone concentration. At a concentration of 80 mg/L (2 MIC), juglone achieved an outstanding corrosion inhibition efficiency of 97.5%, with the corrosion current density (i_corr_) decreasing from 3.16 × 10^−5^ A/cm^2^ to 7.94 × 10^−7^ A/cm^2^.
(3) A dual-action inhibition mechanism is proposed for juglone. At lower concentrations (0.5–1 MIC), the primary role of juglone is antibacterial, reducing the population of corrosive SRB and suppressing biofilm formation. At higher concentrations (2 MIC), in addition to its potent antibacterial effect, juglone chemically reacts with hydrogen sulfide (H_2_S), a corrosive metabolic byproduct of SRB, to form a thiol-containing derivative. This in situ generated derivative adsorbs strongly onto the steel surface, forming a dense and stable protective film that effectively isolates the metal from the corrosive environment.
(4) Surface characterization techniques (SEM, XPS, XRD) provided compelling evidence for the proposed mechanism. The disappearance of FeS peaks and the emergence of characteristic R–SH (thiol) peaks in the XPS spectra confirmed the reaction between juglone and H_2_S. The formation of a protective film was further supported by the emergence of a second time constant in the EIS Bode plots and the requirement of a film resistance (R_f_) in the equivalent circuit model for the 2 MIC system.
(5) This study demonstrates that juglone, synthesized via an improved one-pot method with a high yield of 83.9%, represents a sustainable and cost-effective green alternative to conventional toxic biocides for controlling MIC in marine applications. The utilization of walnut husk waste for juglone production aligns with circular economy principles and offers potential socioeconomic benefits for walnut-producing regions. Future work should focus on elucidating the precise chemical structure of the juglone–H_2_S adduct, quantifying the contribution of the antibacterial and film-forming mechanisms under different environmental conditions, and evaluating the long-term performance of juglone in field applications.

## Data Availability

The original contributions presented in the study are included in the article, further inquiries can be directed to the corresponding authors.

## Supplementary Materials

The following supporting information can be downloaded at: https://www.mdpi.com/article/10.3390/microorganisms14050966/s1, Table S1: Contents of C 1s, O 1s, Fe 2p, and S 2p in the corrosion products of Q235 after immersion in SRB inoculation culture media with different concentrations of Juglone for 14 days; Table S2: Fitting-parameters of potentiodynamic polarization curve and corrosion inhibition efficiency.
