# Supplementary material for: Performance of Juglone as a Natural Extract for Inhibiting SRB-Induced Corrosion of Q235 Steel in Seawater
Source: Microorganisms. 2026 Apr 24;14(5):966. doi: 10.3390/microorganisms14050966 (PMC13209834; doi:10.3390/microorganisms14050966)
Supplement: Supplementary file 1 [file microorganisms-14-00966-s001.zip › microorganisms-4246959-supplementary.pdf]

(atomic fraction / %)

Concentration	C 1s	Fe 2p	S 2p	O1s
**0**	55.17	7.4	4.27	33.17
**0.5*MIC***	44.61	9.63	2.82	42.93
**1*MIC***	35.87	14.62	1.88	47.63

Table S1. Contents of C 1s, O 1s, Fe 2p, and S 2p in the corrosion products of Q235 after immersion in SRB inoculation culture media with different concentrations of Juglone for 14 days

**Condition**	**Ecorr (V vs. SCE)**	**Icorr (A/cm²)**	**βa (mV/dec)**	**βc (mV/dec)**	**η (%)**
SRB	-1.05	3.16 × 10-5	125	-450	-
SRB + 0.5 *MIC*	-1.02	6.31 × 10-6	108	-380	80
SRB + 1 *MIC*	-1	1.58 × 10-6	95	-355	95
SRB + 2 *MIC*	-0.98	7.94 × 10-7	88	-320	97.5

Table S2. Fitting-parameters of potentiodynamic polarization curve and corrosion inhibition efficiency
